# Immunogenicity of Outer Membrane Proteins VirB9-1 and VirB9-2, a Novel Nanovaccine against *Anaplasma marginale*

**DOI:** 10.1371/journal.pone.0154295

**Published:** 2016-04-26

**Authors:** Liang Zhao, Donna Mahony, Antonino S. Cavallaro, Bing Zhang, Jun Zhang, James R. Deringer, Chun-Xia Zhao, Wendy C. Brown, Chengzhong Yu, Neena Mitter, Anton P. J. Middelberg

**Affiliations:** 1 Australian Institute for Bioengineering and Nanotechnology, The University of Queensland, St Lucia, QLD, 4072, Australia; 2 Queensland Alliance for Agriculture and Food Innovation, The University of Queensland, St Lucia, QLD, 4072, Australia; 3 Animal Science, Queensland Department of Agriculture, Fisheries and Forestry, St Lucia, QLD, 4072, Australia; 4 Department of Veterinary Microbiology and Pathology, Washington State University, College of Veterinary Medicine, P.O. Box 647040, Pullman, WA, 99164–7040, United States of America; National University of Singapore, SINGAPORE

## Abstract

*Anaplasma marginale* is the most prevalent tick-borne livestock pathogen and poses a significant threat to cattle industry. In contrast to currently available live blood-derived vaccines against *A*. *marginale*, alternative safer and better-defined subunit vaccines will be of great significance. Two proteins (VirB9-1 and VirB9-2) from the Type IV secretion system of *A*. *marginale* have been shown to induce humoral and cellular immunity. In this study, *Escherichia coli* were used to express VirB9-1 and VirB9-2 proteins. Silica vesicles having a thin wall of 6 nm and pore size of 5.8 nm were used as the carrier and adjuvant to deliver these two antigens both as individual or mixed nano-formulations. High loading capacity was achieved for both proteins, and the mouse immunisation trial with individual as well as mixed nano-formulations showed high levels of antibody titres over 10^7^ and strong T-cell responses. The mixed nano-formulation also stimulated high-level recall responses in bovine T-cell proliferation assays. These results open a promising path towards the development of efficient *A*. *marginale* vaccines and provide better understanding on the role of silica vesicles to deliver multivalent vaccines as mixed nano-formulations able to activate both B-cell and T-cell immunity, for improved animal health.

## Introduction

*Anaplasma marginale* is the most prevalent tick-borne livestock pathogen which causes an infection within cattle, commonly known as ‘Tick Fever’ [1, 2]. The disease results in significant morbidity and mortality in worldwide cattle populations, causing huge economic losses per year estimated to be over $300 million US dollars in the United States, over $100 million in Australia, and approximately $800 million in Latin America [2]. Cattle that recover from acute infection remain persistently infected without clinical signs and serve as reservoirs for biological transmission by ticks [3, 4]. Currently, the commercially-available vaccines are live blood-derived vaccines, which have the potential for transmission of other pathogens and reversion to virulence [4]. Combavac 3 in 1 live tick fever vaccine [5] is one of the marketed vaccines against *A*. *marginale*. It has many disadvantages, such as cold chain for transport and storage, a short shelf life after thawing, and the requirement of multiple doses for vaccination [5]. For these reasons, the currently available vaccines for *A*. *marginale* are considered neither safe nor efficacious [2, 4].

Studies have shown immunisation with purified *A*. *marginale* outer membranes (OM) can induce complete protection against infection by homologous strains [6]. This protection is believed to be associated with CD4^+^ T-lymphocyte-mediated interferon gamma (IFN-γ) activating macrophages and immunoglobulin G2 (IgG2) antibody against outer membrane protein epitopes. However, a vaccine based on the purified OM cannot be cost-effectively manufactured due to the high cost of purification of *A*. *marginale* from erythrocytes and the complex method of purification. A promising way to overcome these problems is to use *A*. *marginale* recombinant proteins as subunit vaccines in combination with the design of novel delivery systems.

Recently, researchers have identified over 20 novel antigenic proteins in a complex *A*. *marginale* OM immunogen by using mass spectrometry and genomic mapping techniques [7]. Among them, proteins VirB9-1 and VirB9-2 of the type IV secretion system (T4SS) were demonstrated to be two of the most promising candidate antigens [7, 8]. The T4SS is a membrane protein complex usually comprising 12 proteins which have been found in many gram-negative bacteria [9, 10]. The function of the T4SS in *A*. *marginale* is transportation of macromolecules, proteins or DNA across the bacterial cell envelope into host cells, which is considered essential for virulence and intracellular survival [8–11]. Targeting and neutralising T4SS proteins with antibodies may adversely affect bacterial survival and dissemination. Studies have shown that proteins VirB9-1 and VirB9-2 together with VirB10 are the most immunogenic of the T4SS proteins [8, 11]. They elicit significant CD4+ T-lymphocyte proliferation, IFN-γ secretion and IgG2 production in outer membrane-immunised cattle, responses that are associated with protective immunity [8, 11]. These proteins that associate to form the outer cap of the T4SS complex are believed to be surface-exposed due to the lack of surface lipopolysaccharide in *A*. *marginale*, and are highly conserved among *A*. *marginale* strains [9–12]. Furthermore, the proteins VirB9-1 and VirB92 are associated in the OM and VirB9-2 can provide T-cell help to produce VirB9-1-specific IgG through linked recognition [8, 12]. Therefore, both proteins are potentially excellent vaccine candidates.

Recombinant proteins alone are often not sufficiently immunogenic, and require adjuvants or carriers in subunit vaccine formulations to enhance the activation of dendritic cells to generate strong antigen specific immune responses [13, 14]. In recent years, many types of nanoparticles have been explored as nanocarriers for vaccine delivery [14–16]. An advantage is nanoparticles' similar size (50–200 nm) to that of cellular components, giving them the ability to enter living cells using the cellular endocytosis mechanism [15]. This allows nanoparticles to improve antigen stability and immunogenicity by presenting antigens very efficiently to antigen presenting cells [16]. In particular, silica-based nanoparticles can be designed into various sizes, shapes, and surface properties suitable for targeted delivery and slow release [14]. Silica nanoparticles are also non-toxic and have excellent biocompatibility [14]. These unique properties make silica nanoparticles excellent nanocarriers for subunit vaccine formulations.

In this study, we have utilised silica vesicles SV-100 [17] to load two *A*. *marginale* protein antigens, VirB9-1 and VirB9-2, that were expressed in *E*. *coli* as soluble and insoluble proteins, respectively. The immunogenicity of each protein and the combination of both proteins were studied in mice, and compared with a conventional adjuvant Quil A (a saponin purified from *Quillaja saponaria* Molina bark). To the best of our knowledge, this is the first time to demonstrate the effect of the combination of two proteins injected as mixed formulation with nanoparticle system on immunogenicity.

## Materials and Methods

### Production of VirB9-1

The coding region of VirB9-1 was amplified from a plasmid containing the VirB9-1 gene [11] by polymerase chain reaction (PCR). The amplified DNA fragments were cloned into pGEX-4T1 glutathione S-transferase (GST) tagged expression vector with the thrombin recognition site sequence mutated into tobacco etch virus (TEV) protease recognition site sequence. The cloning was performed by The University of Queensland’s Protein Expression Facility (PEF, St Lucia, Australia). Purified plasmid DNA was sequenced using ABI BigDye Terminator v3.1 Sequencing (Australian Genome Research Facility (AGRF), Brisbane, Australia).

The plasmid constructs were transformed into *E*. *coli* strain Rosetta (DE3) pLysS. A single colony from the transformation plate was inoculated into 5 mL TB media (12 g/L tryptone, 24 g/L yeast extract, 0.4% (v/v) glycerol, 2.31 g/L KH_2_PO_4_, 12.54 g/L K_2_HPO_4_) and incubated at 30°C on a rotary shaker at 180 rpm for 16 hours. A culture of 800 mL TB media was inoculated with 800 μL of overnight culture and incubated at 37°C, 180 rpm. When the OD600 reached 0.7–0.8, the culture was cooled in a cold water bath prior to induction with 0.2 mM isopropyl β-D-1-thiogalactopyranoside (IPTG) and then incubated at 15°C 180 rpm for 17 hours. Cell pellets were harvested by centrifugation at 6000 *g*, 4°C for 20 minutes and stored at -80°C until further use. All media for culture were supplemented with 50 μg/mL ampicillin and 34 μg/mL chloramphenicol.

VirB9-1 was expressed as a GST-tagged protein and purified using affinity chromatography. The cell pellets were resuspended in L buffer (40 mM Tris-base, 200 mM NaCl, 1 mM ethylenediaminetetraacetic acid (EDTA), 5% (v/v) glycerol, 5 mM dithiothreitol (DTT), pH 8.0) and sonicated with a Branson Sonifier 450 cell disruptor (Branson Ultrasonics Corporation, Connecticut, USA) at output 30 for 4 cycles of 40 seconds. Lysates were centrifuged at 27,000 *g*, 4°C for 20 minutes and the supernatant was filtered through a 0.45 μm filter. The clear filtered lysate was then loaded onto a GST affinity column (GSTrap HP 5 mL, GE Healthcare, UK) pre-equilibrated with five column volumes of L buffer. The column was washed with four column volumes of L buffer to remove unbound contaminants before the GST-tagged VirB9-1 (GST-VirB9-1) protein was eluted with E buffer (40 mM Tris-base, 50 mM NaCl, 1 mM EDTA, 5% (v/v) glycerol, 5 mM DTT, 10 mM glutathione, pH 8.5). Purified GST-tagged VirB9-1 proteins were separated from aggregates and truncations through size-exclusion chromatography (Superdex 200 10/300 GL, GE Healthcare, UK). The fractions were concentrated by spin columns (Vivaspin 2, GE Healthcare, UK), then dialysed against phosphate-buffered saline (PBS, pH 6.5, 300mM NaCl) with a 10,000 MWCO snakeskin dialysis membrane (Thermo Scientific) overnight with minor stirring at 4°C. Endotoxin removal was performed by using Vivapure Q Mini H spin columns (Sartorius Stedim Biotech). Protein integrity was determined by sodium dodecyl sulphate polyacrylamide gel electrophoresis (SDS-PAGE) and the yield was determined by colorimetric assay (BioRad DC protein assay Kit, Hercules, USA).

### Production of VirB9-2

The coding region of VirB9-2 was amplified from a plasmid containing the VirB9-2 gene [11]. The template DNA was amplified using 1 unit of Taq polymerase (NEB Biolabs) and associated buffer (1x), 0.5 μM of each primer; VirB9-2-Fwd: (AAAAACTTGCTTGCGTGC) and VirB9-2-Rev: (GATAAGCACCGTATTCACTAC) and 0.1 mM dNTP. PCR cycling conditions comprised of an initial incubation at 95°C for 5 minutes, followed by 35 cycles at 94°C for 30 seconds, 44°C for 30 seconds and 72°C for 90 seconds. The resultant 820 bp product was ligated into the pET-SUMO vector (Invitrogen, Carlsbad, USA). The ligation products were subsequently transformed into electrocompetent *E*. *coli* strain DH10B (Invitrogen). Positive clones were confirmed by sequencing (AGRF, Brisbane, Australia) and transformed into *E*. *coli* strain BL21 (DE3, Invitrogen) cells for protein expression.

A 2.5 mL overnight culture of *E*. *coli* BL21 (DE3) containing pET-SUMO-VirB9-2 was used to inoculate four 250 mL cultures of LB Miller broth (Amresco, Solon, USA) containing 50 mg/L kanamycin-sulphate (Amresco). These cultures were grown at 37°C to an OD600 between 0.4 to 0.6, then induced with 1 mM IPTG and grown for a further 4 hours. The bacterial pellet was collected by centrifugation at 3,800 *g*, at 4°C for 15 minutes in 4 × 250 mL centrifuge tubes. Total protein was extracted by resuspending bacterial pellets in 50 mL *E*. *coli* lysis buffer (50 mM KPO_4_ phosphate, 400 mM NaCl, 100 mM KCl, 10% (v/v) glycerol, 0.5% (v/v) Triton X-100, 10 mM Imidazole, pH 7.8), with the addition of 12.5 mg Lysozyme and 750 units of Benzonase nuclease (Novagen-Merck, Darmstadt, Germany). The bacterial suspensions were incubated in lysis buffer for 20 minutes with gentle shaking.

The samples were frozen in liquid nitrogen, and thawed at 42°C three times. The resultant solution was centrifuged at 37,000 *g* at 4°C for 15 minutes.

The insoluble protein fraction, containing inclusion-bodies (IB) was purified using BugBuster (Novagen-Merck, Darmstadt, Germany) according to the manufacturers' protocol and as previously described [18]. Purified IB pellets were dissolved in Tris buffer (50 mM Tris, 100 mM DTT, 1% (w/v) SDS, 10% (v/v) glycerol, pH 6.8), followed by incubation at 37°C for 20 minutes. The resulting solubilised protein was dialysed at room temperature, with three buffer changes over 24 hours against PBS (pH 7.4). Protein integrity was determined by SDS-PAGE and yield determined by colorimetric assay. Endotoxin levels were determined by PEF using the Endosafe-PTS Limulus Amebocyte Lysate assay kit.

### Nanoparticle preparation

The silica nanoparticles (SV-100) were synthesised as reported previously [17]. In brief, 0.5 g of EO_39_BO_47_EO_39_ and 0.852 g of Na_2_SO_4_ were dissolved in 30 g of pH 4.7 NaAc-HAc buffer solution ([NaAc] = [HAc] = 0.40 M) to form a homogenous solution under stirring at 10°C. 3.57 mL (3.33 g) of TEOS was added to the above solution with continuous stirring for 24 hours. The reaction mixture was then moved to an autoclave and hydrothermally treated at 100°C for another 24 hours. The as-synthesised samples were collected by filtration and thoroughly washed with deionised water to remove the added salts. The samples were then dried in air. The final products were obtained by calcination at 550°C for 5 hours in air.

### Nanoparticle characterisation

Field-emission scanning electron microscope (FE-SEM) images were obtained using JEOL JSM 7800 operated at 0.6 kV. Transmission electron microscopy (TEM) images were obtained with JEOL 2100 operated at 200 kV. The samples for TEM measurements were prepared by dispersing and drying the powder samples-ethanol dispersion on carbon film on a Cu grid. Nitrogen adsorption-desorption isotherms were measured at 77 K by using a Micromeritics Tristar II system. Samples were degassed at 453 K overnight on a vacuum line prior to the Nitrogen sorption analysis. The total pore volume was calculated from the amount adsorbed at a maximum relative pressure (P/P_0_) of 0.99. The Barrette-Joynere-Halanda (BJH) method was utilised to calculate the entrance size from the desorption branches of the isotherms, and the Brunauere-Emmette-Teller (BET) method was utilised to calculate the specific surface areas. Zeta potential measurements were conducted on a Zetasizer Nano ZS analyser (Malvern Instruments, Worcestershire, UK).

### Adsorption and desorption

The adsorption studies used 2 mg of SV-100, with 1000 μg of GST-VirB9-1 and 1000 μg of SUMO-VirB9-2 in sterile PBS (pH 7.4), respectively. The particle-protein suspension was placed on a Ratek orbital shaker at 4°C and 200 rpm. After 12 hours, a sample of particle-protein slurry (50 μL) was removed and centrifuged at 16,200 g for 1 minute. The supernatants were assessed by colorimetric assay using the BioRad DC protein assay kit (Hercules, USA), and amount of the unbound proteins was determined.

The adsorbed VirB9-1 and VirB9-2 SV-100s were resuspended in 1 mL PBS and incubated at 37°C for 24 hours at 200 rpm. The samples were centrifuged at 16,200 g for 1 minute and the supernatants were analysed by colorimetric assay using the BioRad DC protein assay to determine the amount of desorbed proteins.

### Immunisation

Eight-week-old female C57BL/6J mice were purchased from and housed in the Biological Resource Facility (The University of Queensland, Brisbane, Australia) under specific pathogen-free conditions, with 5 animals per cage in an environmentally controlled area with a cycle of 12 hours of light and 12 hours of darkness. All animal experimental work was reviewed and approved by The University of Queensland Animal Ethics Committee (AEC Approval Number: AE04071). All animals were cared for humanely in accordance with the Australian Code of Practice for the Care and Use of Animals for Scientific Purposes 8^th^ Edition 2013. Eight groups of five mice were immunised with different prototype nanovaccine formulations as shown in Table 1.

**Table 1:** Immunisation groups in mice trial.

Group	Prototype nanovaccine formulation
1	VirB9-1 (50 μg) + Quil A (10 μg)
2	VirB9-1 (50 μg)/SV-100 (250 μg)
3	VirB9-2 (50 μg) + Quil A (10 μg)
4	VirB9-2 (50 μg)/SV-100 (150 μg)
5	VirB9-1 (50 μg) + VirB9-2 (50 μg) + Quil A (10 μg)
6	VirB9-1 (50 μg)/SV-100 (250 μg) + VirB9-2 (50 μg)/SV-100 (150 μg)
7	SV-100 (375 μg) (control)
8	Unimmunised (control)

All doses were administered with 100 μL saline. VirB9-1/SV-100 and VirB9-2/SV-100 refer to VirB9-1 and VirB9-2 adsorbed to SV-100, respectively.

Three subcutaneous injections were given at the tail base on days 0, 21 and 42. All nanovaccine formulations were prepared in sterile injectable saline on the day of the injection. Blood samples were collected by retro-orbital bleeds after anaesthetisation by methoxyfluorane inhalation on days 0, 21 and 35. Final blood samples were collected on day 56 by heart puncture. All mice were euthanized by carbon dioxide inhalation on day 56, spleens were removed and collected. The animals were weighed weekly and monitored three times per week for their health during the study. A score sheet system was used to monitor the mice on their eating, locomotion, behaviour, appearance and weight. If any mice were scored to be ill or moribund, then it would have been euthanized prior the experimental endpoint. All animals remained in good health for the duration of the study with no visible deleterious health effects.

### Enzyme-linked immunosorbent assay (ELISA)

Mouse sera were tested by ELISA in duplicates for the detection of VirB9-1-specific and VirB9-2-specific antibodies. ELISAs were performed by coating microtitre plates (96 well, Nunc, Maxisorb, Roskilde, Denmark) with 50 μL VirB9-1 or VirB9-2 antigen solution (2 ng/μL) in PBS (pH 7.4) overnight at 4°C. The coating solution was removed and the plates were washed once with PBS-T (1× PBS, 0.1% Tween-20, Sigma-Aldrich), followed by blocking with 5% Bovine Serum Albumin (Sigma-Aldrich) and 5% skim milk powder (Fonterra, Auckland, New Zealand) in PBS-T for 1 hour with gentle shaking at room temperature. Plates were washed three times with PBS-T after blocking. Mouse serum samples were diluted from 1:1000 to 1:16,384,000 in 50 μL PBS and each dilution was added to the wells of the blocked plates followed by incubation for 2 hours at room temperature with gentle shaking. The plates were washed three times with PBS-T after incubation, then HRP conjugated polyclonal rabbit anti-mouse IgG antibodies (Sigma-Aldrich) were added to each well at 1:100,000 dilutions in PBS-T, followed by incubation for 1 hour at room temperature with gentle shaking. Plates were washed three times with PBS-T. 100 μL of TMB substrate (Life Technologies) was added to each well and incubated for 6 minutes at RT; 100 μL of 1 N HCl was added to the wells after 6 minutes to stop the chromogenic reaction. The plates were read at 450 nm on the BioTek microplate reader (Winooski, US). Statistical analysis was performed using GraphPad Prism Version 5.03 (GraphPad Software Inc., USA). Comparison between two groups was performed with t test where *p* < 0.05 was considered statistically significant.

### Isolation of murine splenocytes and enzyme-linked immunosorbent spot (ELISPOT) assay

Spleens were aseptically removed and collected from the immunised mice following euthanasia and placed into 5 mL ice cold Dulbecco's modified Eagle's medium (DMEM) supplemented with foetal bovine serum (FBS, 10%), 20 mM HEPES (pH 7.3), 1 M sodium pyruvate, 1 M Glutamax, 100 units/mL penicillin G, 100 μg/mL streptomycin, 0.25 μg/mL Fungizone. Spleens were gently disrupted and passed through a 100 μm nylon mesh (Becton Dickinson, Franklin Lakes, NJ). Spleen cells were washed with 5 mL DMEM and centrifuged at 800 *g*, 4°C for 5 minutes. Cells were then resuspended in 1 mL lysis buffer (0.15 M NH_4_Cl, 10 mM KHCO_3_, 0.1 mM EDTA) for 5 minutes at room temperature. Cell numbers were determined by staining with 0.2% trypan blue. ELISPOT assays were performed by using Mabtech ELISPOT kit (Mabtech, Sweden). ELISPOT plates pre-coated with monoclonal IFN-γ capture antibody were conditioned with complete DMEM medium for 30 minutes at room temperature. Spleen cells from each mouse were seeded at 100,000 cells/well in triplicate into the ELISPOT plates. Cells were incubated in complete DMEM medium at 37°C and 5% CO_2_ for 48 hours in the presence or absence of 10 μg/mL VirB9-1 antigen or VirB9-2 antigen, or the polyclonal activator concavalin A (Con A, 1 μg/mL, Sigma Aldrich) as a positive control. Detection of spots was performed according to manufacturer’s specifications. The ELISPOT plates were read on an ELISPOT reader (Autoimmun Diagnostika, Strassburg, Germany). Results are presented as the mean number of spot forming cells (SFC) per million spleen cells. Statistical analysis was performed using GraphPad Prism Version 5.03. Comparison between the groups was performed with one way ANOVA, and *P* < 0.05 was considered statistically significant.

### *A*. *marginale*-specific T-lymphocyte proliferation assays

Holstein cattle 48422, 48432, and 583 were immunised with *A*. *marginale* St. Maries strain OM as described [8] with the exception that animals 48422 and 48432 were immunised four times at three week intervals with 60 μg OM in saponin, whereas animal 583 was immunised for a prior study with the same antigen dose and adjuvant four times at two-week intervals [8]. Two-week T-cell lines were obtained by stimulating 4x10^6^ peripheral blood mononuclear cells (PBMC) in complete RPMI-1640 medium with 5 μg/mL OM for one week in 1.5 mL volumes in 24-well plates. Cells were harvested and washed in complete RPMI-1640 medium, and viable cells obtained after Ficoll-Hypaque purification, if needed. Viable cells were recultured at 7.5x10^5^ cells per well with 2x10^6^ irradiated autologous PBMC in 1.5 mL complete RPMI-1640 without antigen for one week (resting). Cells were harvested and viable cells were cryopreserved in liquid nitrogen in a mixture of 10% dimethyl sulfoxide‎ (DMSO) in foetal bovine serum for use in proliferation assays.

Proliferation assays were carried out in replicate wells of round-bottomed 96-well plates for 3 days using cryopreserved two-week T-lymphocyte lines from calves 48422, 48432, and 583 as described previously [8]. T-cells (2x10^4^ cells) were cultured in replicate wells in a total volume of 100 μl of complete RPMI-1640 medium containing antigen and 2x10^5^ irradiated autologous PBMC as a source of antigen presenting cells. The VirB9-1 and VirB9-2 proteins were used at a final concentration of 1 and 10 μg/mL in complete RPMI-1640 medium or a mixture of the two at a final protein concentration of 2 and 20 μg/mL. Positive controls include *A*. *marginale* OM used at 1 μg/mL and negative controls included uninfected red blood cell membranes (URBC), and recombinant *Babesia bovis* merozoite surface antigen-1 (MSA-1) used at 1 and 10 μg/mL. Nanoparticles loaded with VirB9-1, VirB9-2, or a mixture of the two were tested at final concentrations of VirB9 proteins of 1 and 10 μg/mL or 2 and 20 μg per mL for the mixture. Empty nanoparticles tested at 2.5, 5.0, 7.5, 10, 25, 50, 75, and 100 μg/mL were used as negative controls. T-cell proliferation was quantified by incorporation of 0.25 μCi/well 3H-thymidine (Dupont, New England Nuclear) during the last 6 hours of culture. The radiolabeled DNA was harvested (Tomtec Cell harvester) on glass filters and the emitted β-particles were counted with liquid scintillation. Results are presented as the mean counts per minute (CPM) +/- 1 SD. The cpm of the different antigens were compared to the cpm for the matching concentration of MSA1 with Boneferroni-Holm (one way ANOVA with posthoc test). Statistically significant T-cell stimulation by an antigen was set at a *P*-value < 0.05.

## Results

### Protein antigens

#### Preparation of VirB9-1

**Fig 1:**
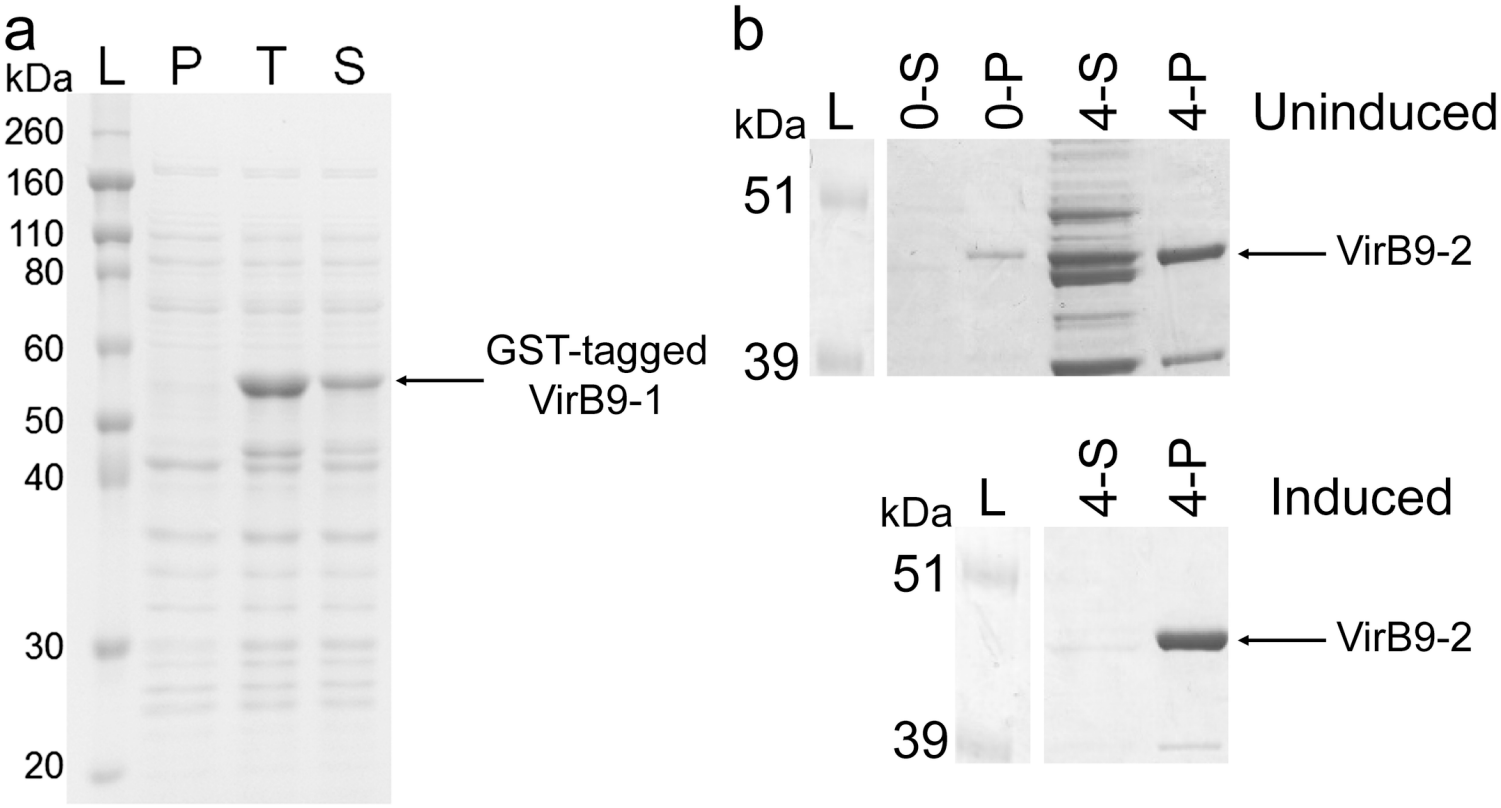
a) Expression of VirB9-1 protein. L: ladder marker; P: uninduced total *E*. *coli* cell lysate; T: induced total *E*. *coli* cell lysate; S: supernatant of *E*. *coli* cell lysate after centrifugation at 27,000 *g*, 4°C for 5 minutes. 6 μL of the ladder was loaded, while the other samples were all 8 μL. GST-tagged VirB9-1 protein is approximately 56 kDa as labelled. b) Expression of VirB9-2 protein. Top: uninduced sample; Bottom: induced sample. L: ladder marker; S: soluble fraction; P: pellet fraction; 0-S: 0 h supernatant; 0-P 0 h pellet; 4-S: 4 h supernatant; 4-P: 4 h pellet. VirB9-2 protein is approximately 44 kDa as labelled.

Our laboratory has developed a simple and low cost technology for high-level expression of soluble VirB9-1 protein in *E*. *coli* using the GST tag [19]. SDS-PAGE analysis (Fig 1a) shows the expression result for the VirB9-1-GST construct, demonstrating its high expression yield with a dominant band at approximately 56 kDa, corresponding to the predicted molecular weight of the construct. By estimate, the total expression yield of VirB9-1 is 70 mg/L of culture for a final culture OD600nm of approximately 3.5. Fig 1 also shows that VirB9-1 had a good solubility of about 50% under pH 8.0 buffer conditions (according to BIO-RAD Image Lab^©^ software). The solubility of VirB9-1 can be further increased to over to 90% using pH 9.0 buffer (data not shown). However, high pH is not suitable for downstream purification as the GST affinity column optimally works in the range of pH 6.5–8.0, therefore pH 8.0 buffer was chosen for protein purification.

**Fig 2:**
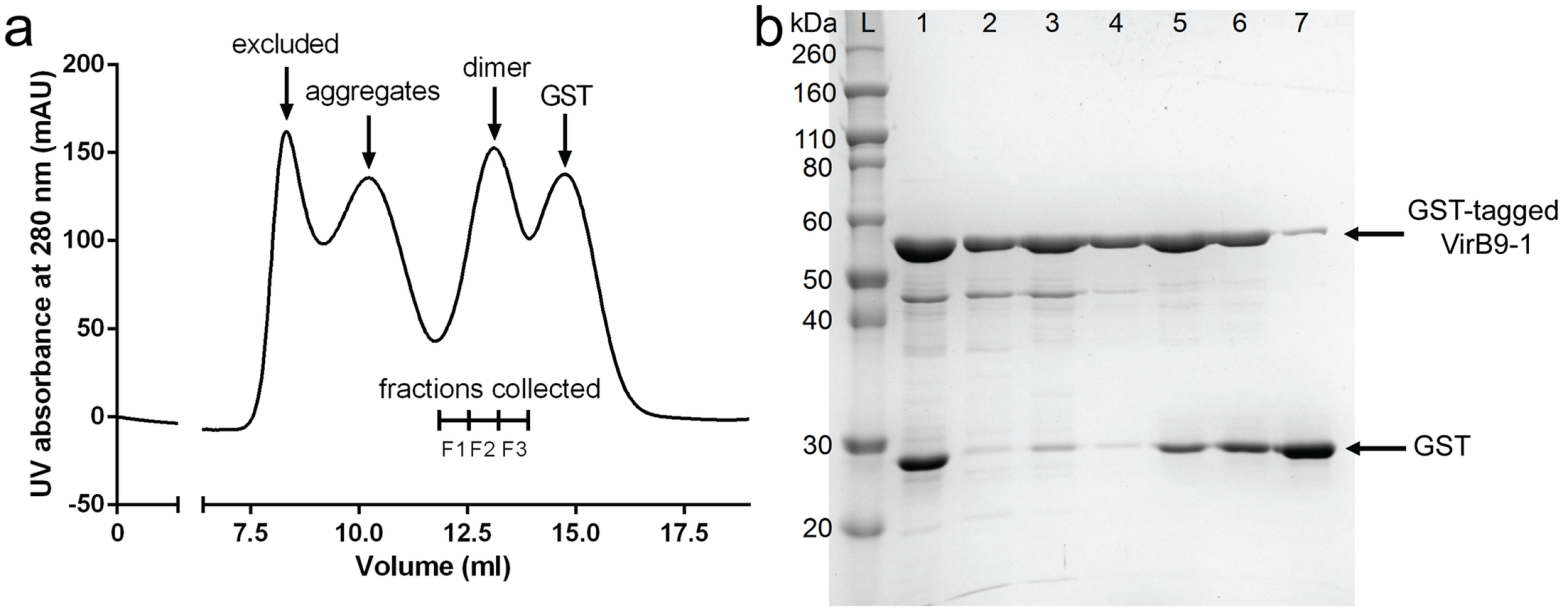
a) Size-exclusion chromatogram of GST-tagged VirB9-1. GST-VirB9-1 dimers were separated from aggregates and GST in size exclusion chromatography pre-equilibrated with L buffer. b) SDS-PAGE analysis of fractions from size-exclusion chromatography. L: ladder marker; 1: fraction from GST affinity chromatograph; 2: excluded peak; 3: aggregates peak; 4: F1 of dimer peak; 5: F2 of dimer peak; 6: F3 of dimer peak; 7: GST peak.

GST-VirB9-1 protein was purified using a two-step chromatography method, by first passing supernatant of the cell lysis after sonication through a GST affinity column, followed by a size exclusion column purification step. Fig 2 shows the size exclusion purification results, where four peaks were of approximately 700, 400, 110 and 55 kDa were obtained (the molecule weights were estimated based on the manufacturer’s instructions of the Superdex 200 10/300 GL column). The components of these four peaks were further analysed using the SDS-PAGE (Fig 2b), and were identified to be large GST-VirB9-1 soluble aggregates (Fig 2b lane 2), small GST-VirB9-1 soluble aggregates (Fig 2b lane 3), GST-VirB9-1 dimers (Fig 2b lane 4–6), and GST dimers (Fig 2b lane 7), respectively. The dimer peak including three fractions (F1, F2 and F3) was collected as the protein antigen. F1 fraction (Fig 2b, lane 4) contains mainly GST-VirB9-1 with the highest purity. F2 and F3 fractions (Fig 2b, lanes 4 and 5) contain not only VirB9-1 but also a 27 kDa GST protein, which confirmed by mass spectrometry and western blot analysis. It is most likely truncated from the GST fusion protein during expression. F1, F2 and F3 fractions were all collected to achieve a high yield of about 2 mg/L of culture of the GST-VirB9-1 protein.

#### Preparation of VirB9-2

**Fig 3:**
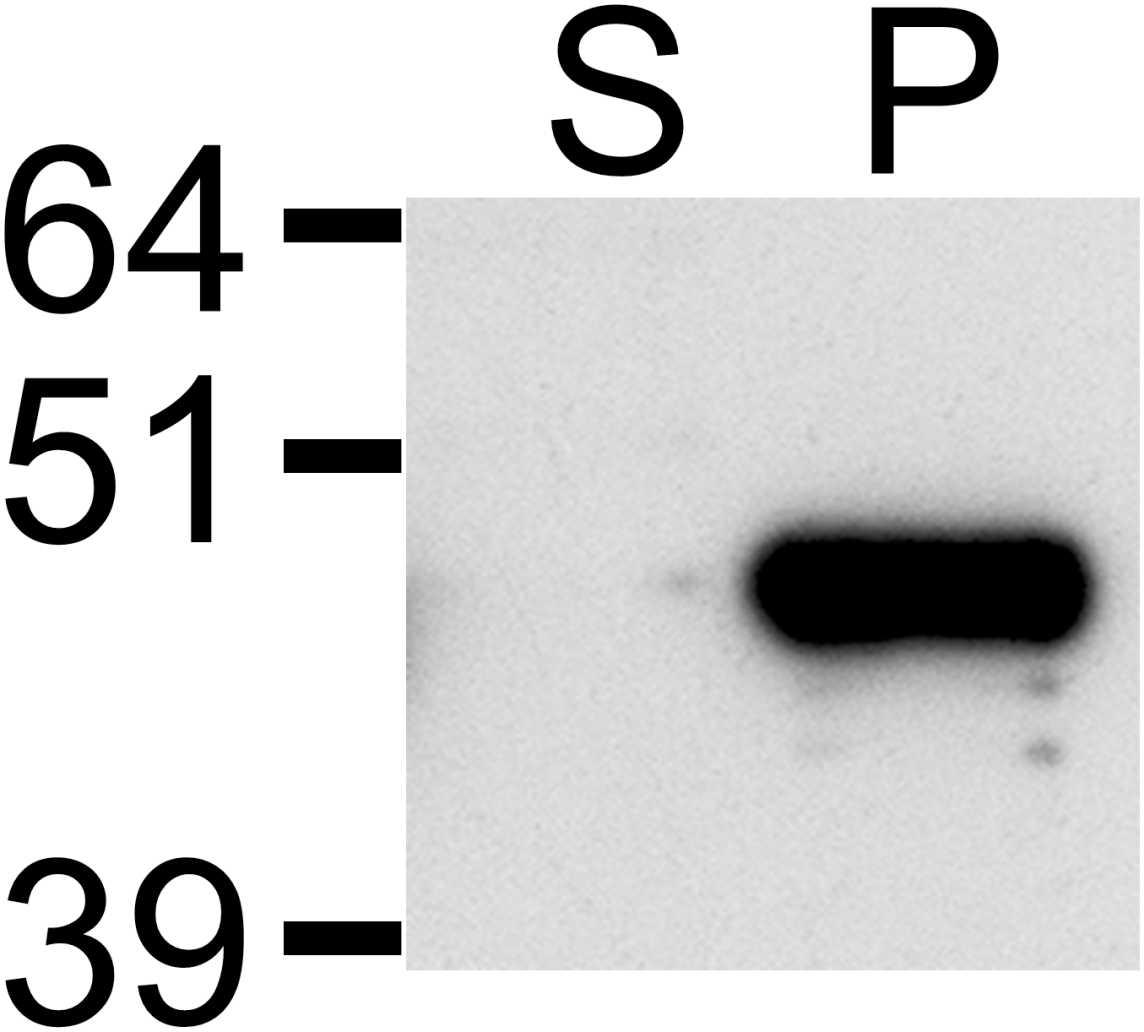
Western blot analysis of pET-SUMO-VirB9-2 expression. Purified protein was separated on a SDS-PAGE gel, transferred to nitrocellulose and probed with Anti-His antibodies. Supernatant (S) and pellet (P) fractions show that the protein is expressed as insoluble inclusion bodies.

Our initial expression studies showed high expression of VirB9-2 at 4 hours post induction with IPTG, as inclusion bodies (Fig 1b). The fusion protein encoded by pET-SUMO-VirB9-2 has a hypothetical MW of 43.9 kDa which is consistent with the western blot analysis (Fig 3). The insoluble protein was solubilised using a DTT buffer followed by dialysis into PBS, resulting in a pure and soluble protein. Following dialysis, the final yield of VirB9-2 was 15 mg/L of culture.

### Silica vesicles as nanocarriers and adjuvants

#### Characterisation of SV-100

**Fig 4:**
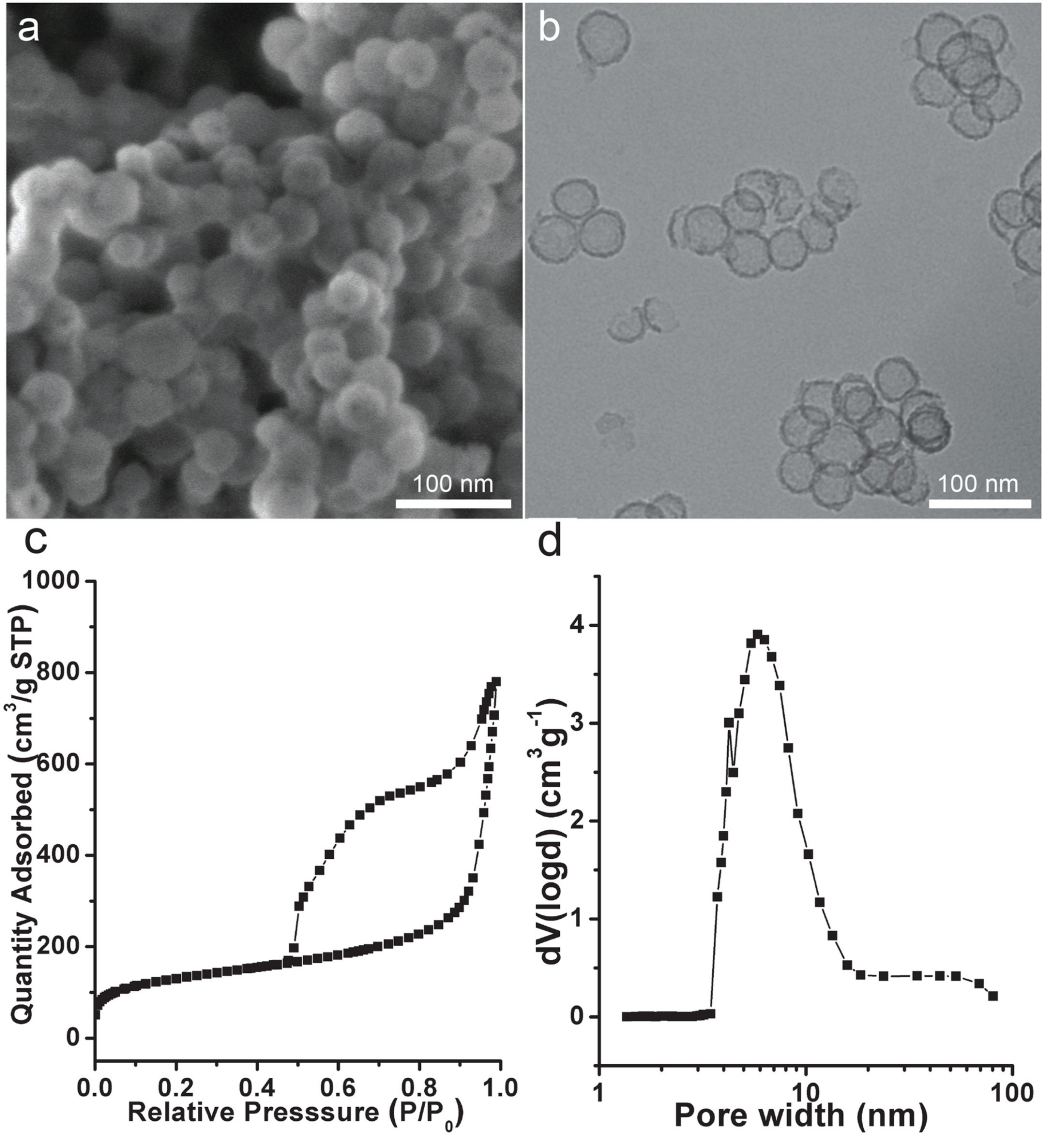
a) FE-SEM and b) TEM image of SV-100; c) Nitrogen sorption isotherm of SV-100; d) Barrett- Joyner -Halanda pore size distribution curve calculated from the desorption branch of SV-100.

The SV-100 silica vesicles have a hollow spherical structure with uniform particles of ~50 nm size and wall thickness of ~6 nm, as measured by the SEM and TEM analyses (Fig 4a and 4b). The nitrogen sorption analysis of SV-100 shows representative type IV isotherms with type H3 hysteresis loop (Fig 4c). The adsorption branch shows major capillary condensation step at relative pressure (P/P_0_) of ~ 0.9. The average pore size, or entrance size, was calculated by the Barrett-Joyner-Halanda (BJH) method from the desorption branch to be 5.8 nm (Fig 4d), suitable for GST-VirB9-1 protein and SUMO-VirB9-2 protein loading. The Brunauer-Emmett-Teller (BET) surface area of SV-100 is 487 m^2^/g, the total pore volume is 1.31 cm^3^/g, and the Zeta potential is -18.1 mV (Table 2).

**Table 2:** Structural information from Nitrogen sorption results of SV-100.

Name	BET surface area (m^2^/g)	Total pore volume (cm^3^/g)	Entrance size (nm)	Zeta potential (mV)
SV-100	487	1.31	5.8	-18.1

**Fig 5:**
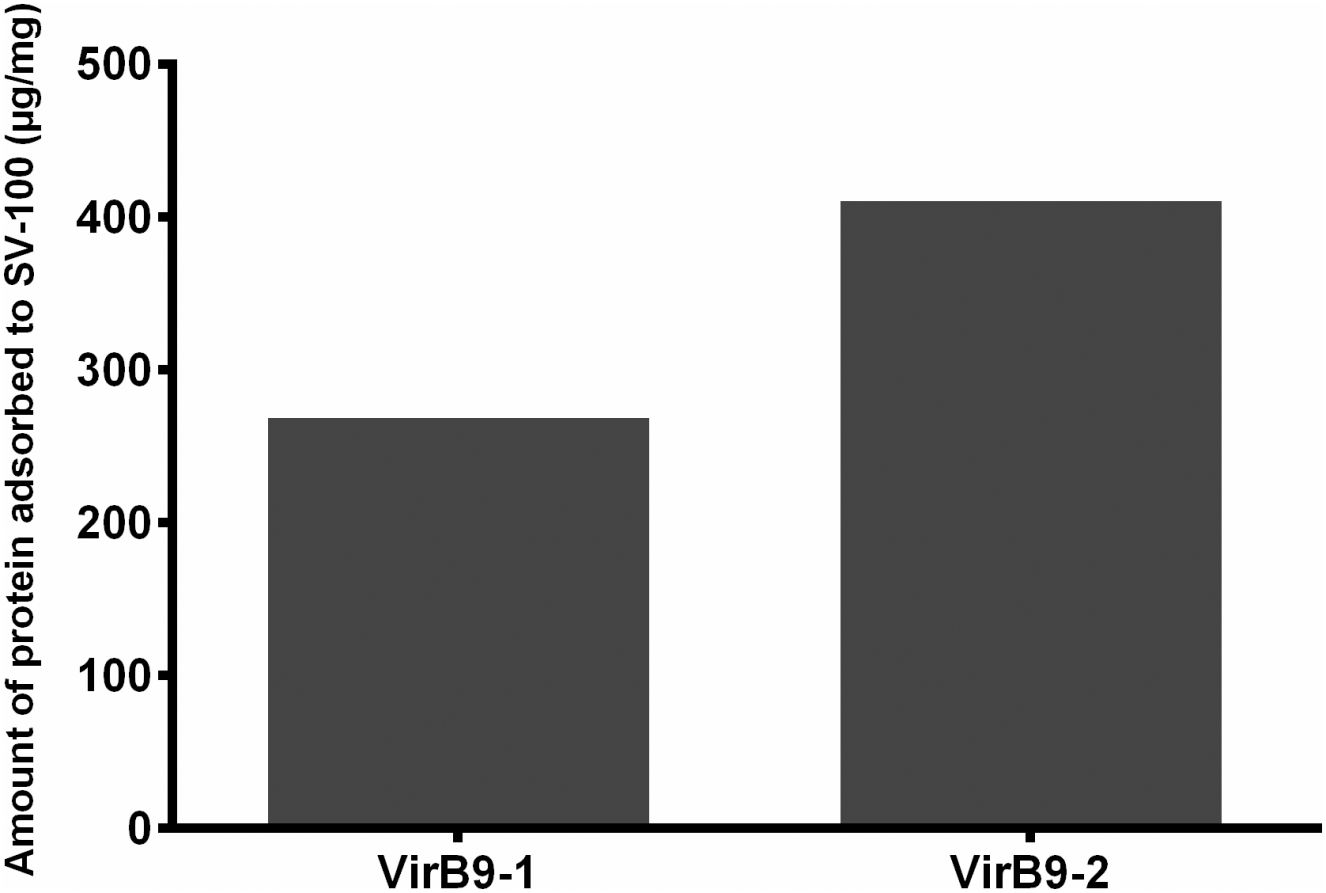
Adsorption amount of VirB9-1 and VirB9-2 proteins onto SV-100 nanoparticles. Data determined by protein assay.

The SV-100 silica vesicles were tested for adsorption capabilities of GST-VirB9-1 and SUMO-VirB9-2 proteins in PBS at 4°C. Fig 5 shows that SV-100 particles have an adsorption for GST-VirB9-1 of 268 μg/mg particle and for SUMO-VirB9-2 of 410 μg/mg particle. This demonstrates the high loading capacity of SV-100 particles by virtue of the hollow structure. The desorption of the VirB9-1 and VirB9-2 loaded SV-100 particles was the same as previously reported [13], there was no desorption of the two protein in PBS at 37°C.

### Immunisation Studies

#### ELISA results

**Fig 6:**
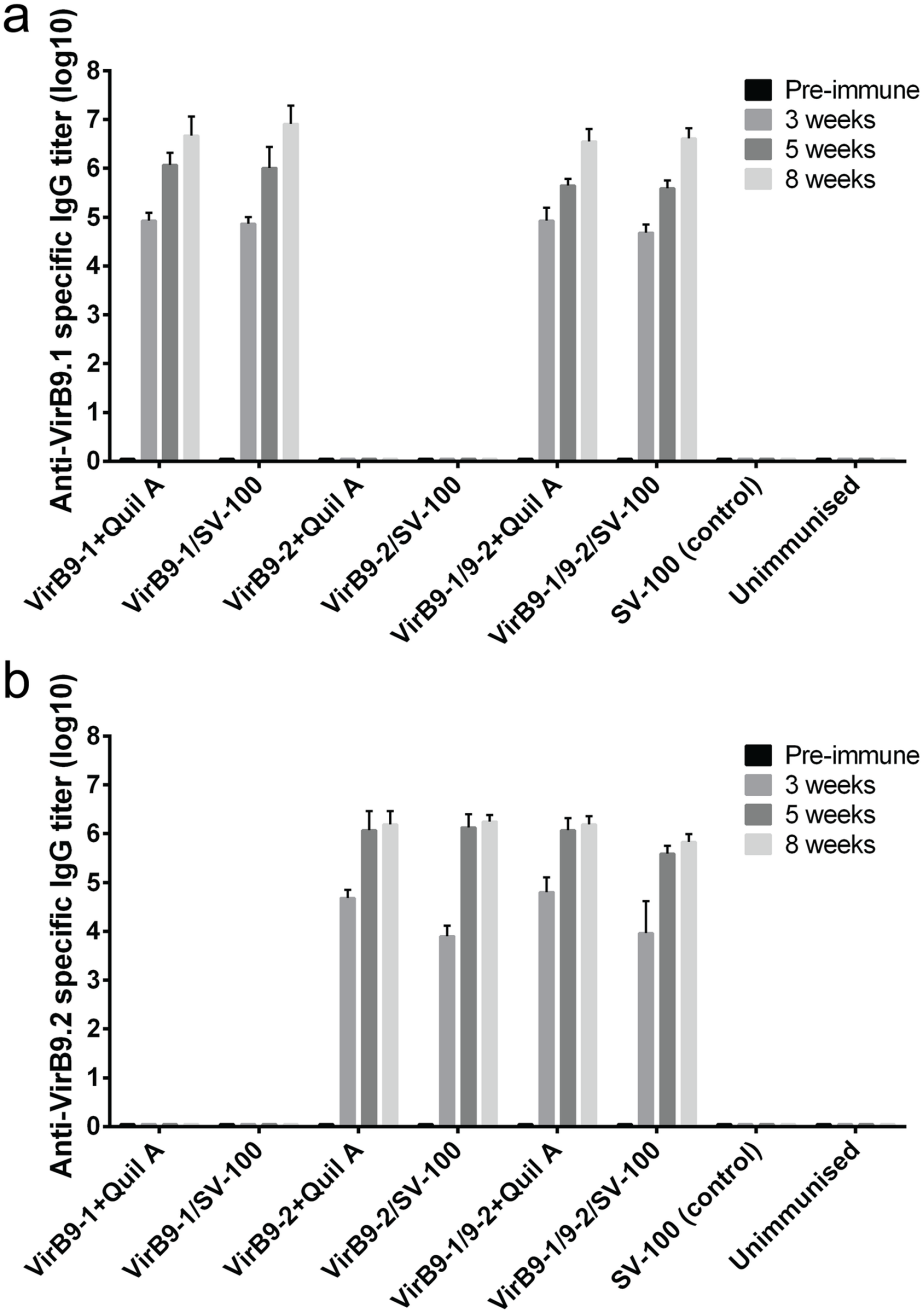
a) VirB9-1-specific antibody end point titres in C57BL/6J mice for different vaccine formulation groups. b) VirB9-2-specific antibody end point titres in C57BL/6J mice for different vaccine formulation groups.

The immune responses of the VirB9-1 and VirB9-2 prototype nanovaccine formulations were investigated using C57BL/6J female mice. Eight groups of five mice received three subcutaneous immunisations of the various formulations (Table 1) at three-week intervals. The endotoxin level of the protein antigens used were 104 EU/mL for VirB9-1 and 115 EU/mL for VirB9-2, equivalent to 3.64 EU per 50 μg injected dose of VirB9-1 and 2.68 EU per 50 μg dose of VirB9-2. The level of endotoxin present in VirB9-1 and VirB9-2 was within safe levels as shown by Schadlich et al [20]. We compared the SV-100 formulation against a conventional adjuvant Quil A, which has been widely used in animal vaccine studies [21]. The sera collected from the immunised mice were analysed by both anti-VirB9-1 and anti-VirB9-2 ELISA to detect the total IgG responses. As both antigens may be required to achieve protective immunity, we not only included each single antigen with Quil A or silica vesicles, but also designed two groups which combined VirB9-1 and VirB9-2 into one mixed formulation to investigate the interaction between them. Fig 6a shows that the VirB9-1-specific IgG response of the VirB9-1/SV-100 group was comparable to that of the VirB9-1 plus Quil A group with a titre of 10^5^, and no significant difference (*P* < 0.05) was observed. After three injections, the VirB9-1/SV-100 group demonstrated an antibody titre of 10^7^. For the VirB9-2-specific IgG response of the VirB9-2 plus Quil A group and VirB9-2/SV-100 group, there were no significant differences (*P* < 0.05) observed between the two groups. After three injections, the overall antibody responses showed titres of over 10^5^ (Fig 6b). The groups receiving mixed formulation (VirB9-1 and VirB9-2 loaded separately to SV-100 and mixed before injection) showed both strong VirB9-1-specific antibody responses and strong VirB9-2-specific antibody responses, with average endpoint titres of 10^6^ after three injections. The titres of the double antigen groups showed no significant difference (p < 0.05) to the single antigen groups. The negative control group receiving only SV-100 as control and the unimmunised group showed no VirB9-1-specific or VirB9-2-specific antibody responses. A Quil A negative control group was not included in this study, for Quil A by itself without antigens does not induce specific antibody responses as reported previously [22].

#### ELISPOT results

**Fig 7:**
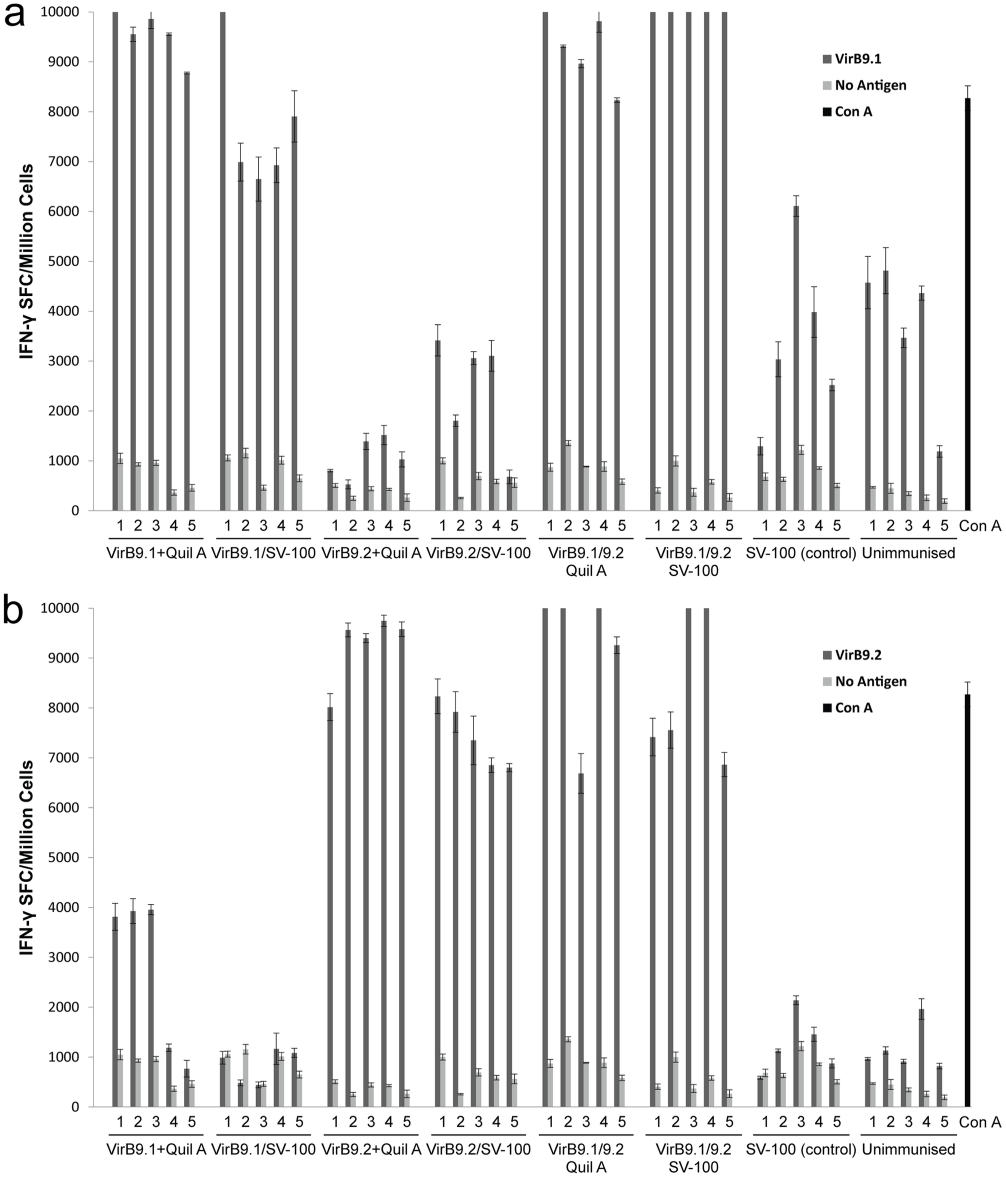
a) Detection of VirB9-1-specific IFN-γ secretion by ELISPOT assay of murine splenocytes from immunised mice. b) Detection of VirB9-2-specific IFN-γ secretion by ELISPOT assay of murine splenocytes from immunised mice. 1 to 5 are the individual mice in each group. See S1 Table for statistical analysis.

In addition to antibody-mediated immunity, cell-mediated immunity in response to the nanovaccine formulations was investigated. Splenocytes were analysed by ELISPOT assay to determine the T-helper type 1 (Th1) cell mediated IFN-γ responses to VirB9-1 and VirB9-2 antigen. All mice in the VirB9-1 plus Quil A group, VirB9-1/SV-100 group, VirB9-1/9-2 plus Quil A group, and VirB9-1/9-2/SV-100 group showed very high cell-mediated immune responses to VirB9-1 antigen, as indicated by the number of cells producing IFN-γ, ranging from 6,650 to over 10,000 SFC/million cells (extending beyond the axis of the graph) (Fig 7a). These four groups all showed significant differences (*P* < 0.0001) to the two negative control groups (SV-100 and unimmunised) and the two groups that only received VirB9-2 antigen. The VirB9-1/9-2/SV-100 group showed the highest response where all five animals had over 10,000 IFN-γ SFC/million spleen cells, indicating a very strong cell-mediated immune response. A significant difference (*P* < 0.05) was observed when comparing the VirB9-1/9-2/SV-100 group to the VirB9-1/SV-100 group, but no significant difference (*P* < 0.05) was observed when comparing to the VirB9-1 plus Quil A group and the VirB9-1/9-2 plus Quil A group. The VirB9-2 plus Quil A group, the VirB9-2/SV-100 group, and the two negative control groups only produced background IFN-γ response to VirB9-1 antigen, much lower than the positive control Con A. Fig 7b shows that all mice in VirB9-2 plus Quil A group, VirB9-2/SV-100 group, VirB9-1/9-2 plus Quil A group and VirB9-1/9-2 SV-100 group induced high cell-mediated immune responses to VirB9-2 antigen, as indicated by the number of cells producing IFN-γ, ranging from 6,686 to over 10,000 SFC/million spleen cells (extending beyond the axis of the graph). These four groups all showed significant differences (*P* < 0.0001) when compared to the two negative control groups and the two groups that only received VirB9-1 antigen, but no significant differences (*P* < 0.05) were observed when comparing them to each other. The background IFN-γ response to VirB9-2 antigen was lower than that to VirB9-1, only a few animals in the two negative control groups and the two groups that only received VirB9-1 antigen had an obvious background response. The detailed statistical analysis of each group can be seen in S1 Table.

#### T-lymphocyte proliferation assay results

**Fig 8:**
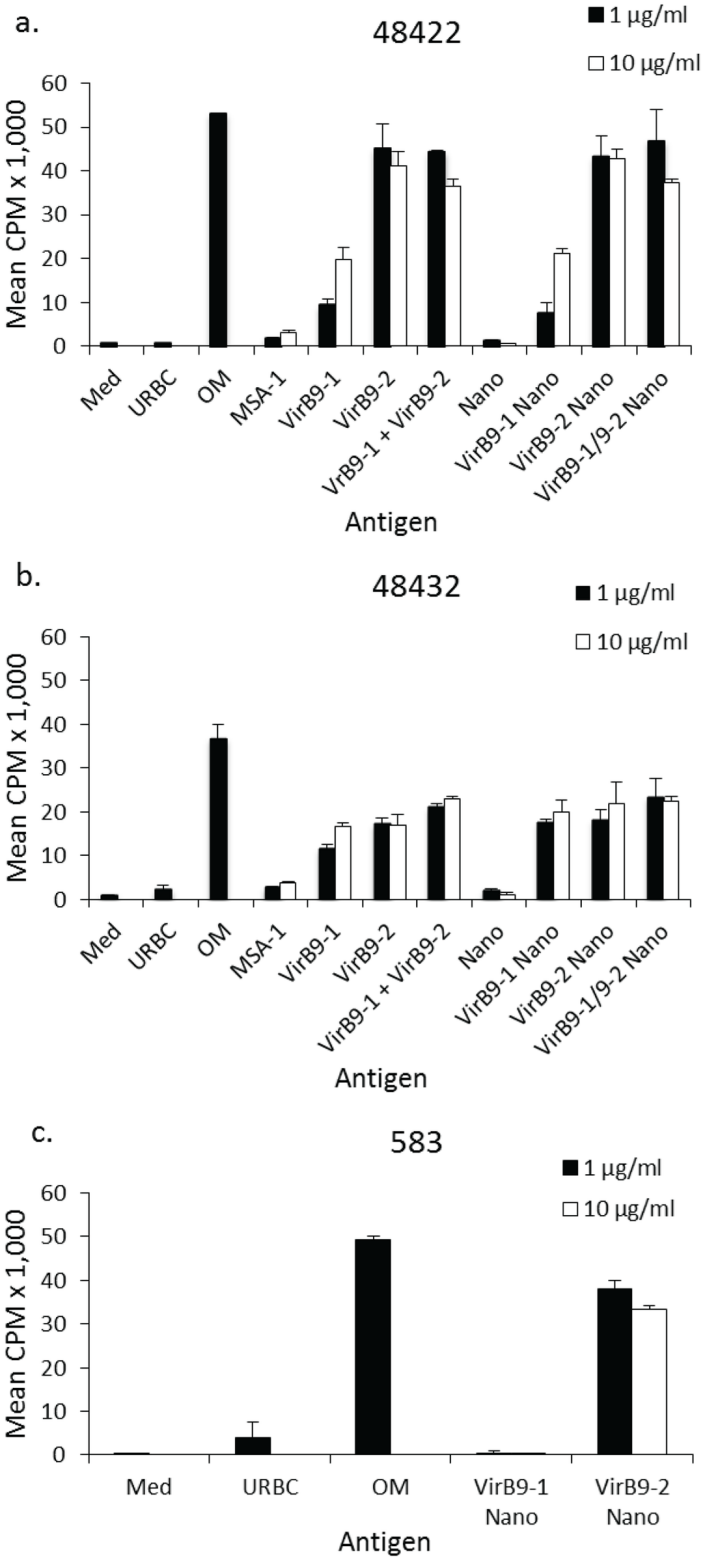
Two-week T-cell lines established from *A*. *marginale* OM-vaccinated calves proliferate in response to VirB9-1 and/or VirB9-2 loaded nanoparticles. T-cells from cattle 48422 (a), 48432 (b) were tested for proliferation to 1 (black bars) μg/mL URBC and purified OM, and to 1 (black bars) and 10 (white bars) μg/mL recombinant *B*. *bovis* MSA-1, and *A*. *marginale* VirB9-1 and VirB9-2, and VirB9-1 and VirB9-2 loaded onto SV-100 nanoparticles. A mixture of VirB9-1 plus VirB9-2 and VirB9-1-SV-100 plus VirB9-2-SV-100 was also used. SV-100 tested at final concentrations of 5 and 50 μg/mL are shown. A two-week T-cell line from animal 583 (c) was tested with VirB9-1 and VirB9-2 loaded nanoparticles to rule out nonspecific stimulation by the *E*. *coli*-expressed protein, as this animal does not respond to VirB9-1.

Both VirB9-1 and VirB9-2 stimulated recall responses in OM-immunised calves 48422 and 48432 (Fig 8). VirB9-2 stimulated slightly higher T-cell proliferation than VirB9-1. There was no cumulative effect in combining the antigens. For animal 48422 (Fig 8a), there was no significant difference observed between VirB9-2 alone and the combination of VirB9-1 and VirB9-2 treatment. However, a significant difference was observed between VirB9-1 and VirB9-2 combination group and VirB9-1 treatment only (*P* < 0.0001 for 1 μg/mL and *P* = 0.0009 for 10 μg/mL). In contrast, animal 48432 (Fig 8b) showed a significant response when comparing the combination of VirB9-1 and VirB9-2 to VirB9-1 (*P* = 0.0004 for 1 μg/mL and *P* = 0.0002 for 10 μg/mL) and to VirB9-2 (*P* = 0.016 for 1 μg/mL and *P* = 0.01 for 10 μg/mL). The responses to VirB9-1 and VirB9-2-loaded SV-100 either alone or in combination were similar to the responses induced by the recombinant proteins for both animals, and there was no response to any concentration of empty SV-100 tested (data not shown and Fig 8a and 8b). Animal 583 did not respond to VirB9-1 but had a significant T-cell response to VirB9-2 using both 1 and 10 μg/mL antigen-loaded nanoparticles (Fig 8c). These data are consistent with previous studies using VirB9-1 and VirB9-2 expressed from the pBAD/TOPO ThioFusion vector [8] and in peptide mapping studies [12], where animal 583 never responded to VirB9-1 but had strong responses to VirB9-2. The lack of response to recombinant MSA-1 for animals 48422 and 48432, together with the predicted lack of response of animal 583 to recombinant VirB9-1 show there is no nonspecific proliferation to the *E*. *coli*-expressed fusion proteins.

## Discussion

VirB9-1 and VirB9-2 are OM proteins of *A*. *marginale*. Due to their intrinsic properties, they are generally difficult to express at high solubility and yield. In this paper we have successfully produced high yielding soluble VirB9-1 and VirB9-2 utilising two different expression and purification systems.

Previous expression studies of VirB9-1 using FLAG-tag or His-tag have resulted in insoluble proteins which were purified using denaturing methods [8, 11, 12, 23]. However, for vaccination, soluble proteins as antigens have great potential to include native structural elements that may be able to induce protective immunity. Therefore, it is essential to develop an approach for producing soluble VirB9-1 proteins that present the key B-cell epitopes that rely on three-dimensional structures. Thus, in this study a VirB9-1-GST fusion protein was designed and expressed in *E*. *coli*, as the use of GST fusion tags can simplify downstream purification, improving overall yield and solubility of the target protein. This is the first time that the VirB9-1 protein has been expressed in *E*. *coli* at high yield and high solubility [11, 23]. The yield is about 70 mg/L of culture after low-density shake-flask expression and about 2 mg/L of culture after purification.

Because of the presence of the GST tag, GST-VirB9-1 proteins can form as a soluble dimer structure with high purity, which is similar to the dimerisation reported previously in aqueous solutions [24]. It was also previously observed that *E*. *coil* expressed recombinant VirB9-1 and native VirB9-1 naturally form dimers [8]. Therefore, after size-exclusion chromatography, the GST-VirB9-1 peak appeared at about 110 kDa, whereas the size of the protein dimer is 112 kDa. The GST tag was not cleaved to simplify the preparation method and reduce the cost of purification by not using expensive enzymes for cleavage. Furthermore, the GST tag may also provide some level of adjuvanting effect [25].

VirB9-2 contains linear T-cell epitopes [12] so high expression, even as an insoluble protein could refold and retain the linear epitopes1 Our previous work has shown pET-SUMO has high expression and we have previously used this system to express optiE2 [18]. Similarly pET-SUMO-VirB9-2 was found to be expressed as insoluble inclusion bodies, and following refolding produced soluble VirB9-2 of suitable quality.

Silica nanoparticles have been established as excellent nanocarriers for protein antigen delivery and subsequent induction of antibody and cell-mediated immune responses in mice [13, 26, 27] and sheep [28], for their attractive properties, such as tuneable size and structure, various surface modifications, and excellent biocompatibility [14, 29]. The next generation SV-100 type silica vesicles with increased protein adsorption capacities [17] have been shown to be potent adjuvants, inducing high levels of both antibody and cell-mediated immune responses in mice with no detrimental effects [13].

We have developed a facile method to synthesise the SV-100 nanoparticles with precise control of both the particle size and the entrance size. The 50 nm size of SV-100 nanoparticles is very efficient for uptake by dendritic cells, and the spherical shape similar to a virus also enhances higher antibody response when compared with other shapes of similar size [14]. Entrance size is an important factor for protein loading, studies show that silica vesicles with an entrance size close the size of the target protein had the highest loading capacity [17]. SV-100 nanoparticle has an entrance size of 5.8 nm, which is close to the size of the two proteins. Both GST-VirB9-1 and SUMO-VirB9-2 achieved high adsorption to SV-100 nanoparticles (Fig 5). The adsorption of VirB9-2 was higher, possibly due to its smaller size than VirB9-1 (44 kDa compared to 56 kDa), enabling the smaller protein to have a better chance of entering the SV-100 nanoparticle.

No desorption was observed from the protein loaded SV-100 nanoparticles in PBS at 37°C. This is likely because the majority of protein was entrapped inside the hollow structures instead of adsorbing at the outer interface. The strong binding also suggests the protein-loaded SV-100 nanoparticles have a good chance of being taken up by dendritic cells for antigen presentation.

Here, using VirB9-1 or VirB9-2 bound separately to SV-100 nanoparticles, we have demonstrated the versatility of the system by demonstrating, to the best of our knowledge for the first time, that it is possible to induce discrete immune responses using a mixed nano-formulation. The mice were injected subcutaneously three times with 50 μg of either VirB9-1 or VirB9-2 adsorbed to SV-100 nanoparticle. The adsorption capacities differed for the two proteins with 53% higher adsorption for VirB9.2 (Fig 5), so that the dose of the injected protein was kept constant across all eight of the groups at a 50 μg dose.

The total IgG responses over time were measured by VirB9-1 and VirB9-2-specific ELISAs and we demonstrated the development of VirB-specific antibodies three weeks after a single injection for both VirB9-1 (Fig 6a) and VirB9-2 (Fig 6b). The antibody titre was lower with average end-point titre of 10^4^ for VirB9.2 compared to 10^5^ for VirB9.1 after a one injection. This could be due to 150 μg SV-100 nanoparticles in the VirB9.2 injection doses compared to 250 μg in the VirB9.1 SV-100 nanoparticle injection doses. This seems to be the case since the level of responses for the groups injected with VirB9-1 Quil A and VirB9-2 Quil A are very similar for both VirB9.1 and VirB9.2. With subsequent injections there is very little difference in the titre levels for VirB9-1 and VirB9-2 and showed that SV-100 nanoparticles act as a comparable adjuvant to Quil A. Whereas Quil A retains a certain level of toxicity and undesirable side effects [30, 31] it is likely that SV-100 nanoparticles will have a lower toxicity profile and better safety [13].

The combination of mixed nano-formulation did not interfere with the VirB9-1- or VirB9-2- specific antibody levels. In addition, as shown previously [12] there was no cross reaction between the two antigens and animals receiving only VirB9-1 showed no response to VirB9-2 and vice versa.

The cell-mediated immune responses were measured by VirB9-1 and VirB9-2-specific IFN- γ ELISPOT. Results show that antigen-loaded SV-100 nanoparticles can induce high levels cell-mediated immunity similar to antigens administered with Quil A, resulting in over 10,000 SFC/million splenocytes. The mixed nano-formulation groups had high numbers of IFN-γ SFC to both VirB9-1 and VirB9-2 antigens, indicating that there was no interference between the two antigens and that they can be injected together in a mixed formulation. The VirB9-1-specific cell-mediated immune responses for the mixed nano-formulation group were even significantly higher than when the antigen was injected singly with SV-100 nanoparticles (Fig 7a). These results show that designing an *A*. *marginale* vaccine using both VirB9-1 and VirB9-2 as antigen is not only feasible, but also may induce better cell-mediated immune responses than using a single antigen. The IFN-γ responses of some mice from the control groups to VirB9-1 antigen were slightly high. This may be due to the higher endotoxin level of the VirB9-1 stimuli (0.15 EU per well) than that of the VirB9-2 stimuli (0.11 EU per well) used in the ELISPOT assay. A higher endotoxin level stimulates the cells and induced higher IFN-γ responses [32].

The bovine T-lymphocyte proliferation assay results further confirmed the benefits of using a mixed nano-formulation to generate cell-mediated immunity. In animal 48422 where VirB9-2 stimulated a much higher T-cell proliferative response than VirB9-1, the mixed formulation achieved an equivalently high response (Fig 8a). In animal 48432 where VirB9-2 stimulated a slightly higher T-cell proliferative response than VirB9-1, the mixed formulation induced an even higher response (Fig 8b).

## Conclusions

This study investigated the immunogenicity of combined VirB9-1 and VirB9-2 antigens in formulation with specially designed silica vesicles. Our findings showed that the combined antigen formulation can induce an equivalent level of antibody and better cell-mediated immune responses (IFN-γ SFC, T-lymphocyte proliferation) than single antigen formulations. This result of combining VirB9-1 and VirB9-2 opens a possible pathway towards developing a new generation of subunit vaccines against *A*. *marginale* using recombinant protein antigens. Furthermore, SV-100 nanoparticles can induce high levels of IgG and cell-mediated immune responses that are comparable to those induced by Quil A, yet are advantageous over Quil A because they are non-toxic and of low cost. These findings can provide a better understanding of the adjuvanting effect of silica nanoparticles and may lead to improvements in future animal vaccine formulations.

## Supporting Information

S1 Table Statistical analysis of ELISPOT results. a. VirB9-1-specific ELISPOT assay results: *, p < 0.05; ****, p < 0.0001; ns = not significant. b. VirB9-2-specific ELISPOT assay results: ****, p < 0.0001; ns = not significant.
(DOCX)
