# Supplementary material for: Immunogenicity of Outer Membrane Proteins VirB9-1 and VirB9-2, a Novel Nanovaccine against Anaplasma marginale
Source: PLoS One. 2016 Apr 26;11(4):e0154295. doi: 10.1371/journal.pone.0154295 (PMC4846087; doi:10.1371/journal.pone.0154295)
Supplement: S1 Table — a. VirB9-1-specific ELISPOT assay results: *, p < 0.05; ****, p < 0.0001; ns = not significant. b. VirB9-2-specific ELISPOT assay results: ****, p < 0.0001; ns = not significant. (DOCX) [file pone.0154295.s001.docx]

**Supporting information**

**­­Immunogenicity of outer membrane proteins VirB9-1 and VirB9-2, a novel nanovaccine against *Anaplasma marginale***

Liang Zhao^1*^, Donna Mahony^2*^, Antonino S. Cavallaro^2^, Bing Zhang^3^, Jun Zhang^1^, James R. Deringer^4^, Chun-Xia Zhao^1^, Wendy C. Brown^4^, Chengzhong Yu^1^, Neena Mitter^2†^, Anton P.J. Middelberg^1†^

^1^ Australian Institute for Bioengineering and Nanotechnology, The University of Queensland, St Lucia, QLD 4072, Australia.

^2^ Queensland Alliance for Agriculture and Food Innovation, The University of Queensland, St Lucia, QLD 4072, Australia.

^3^ Animal Science, Queensland Department of Agriculture, Fisheries and Forestry, St Lucia, QLD 4072, Australia.

^4^ Department of Veterinary Microbiology and Pathology, Washington State University, College of Veterinary Medicine, P.O. Box 647040, Pullman, WA 99164-7040, USA.

^*^ These authors contributed equally.

^†^ Corresponding authors: E-mail addresses: n.mitter@uq.edu.au (N. Mitter); a.middelberg@uq.edu.au (A. P. J. Middelberg).

| **Groups** | VirB9.1+Quil A | VirB9.1/SV-100 | VirB9.2+Quil A | VirB9.2/SV-100 | VirB9.1/9.2+Quil A | VirB9.1/9.2/SV-100 | SV-100 | Unimmunised |
| --- | --- | --- | --- | --- | --- | --- | --- | --- |
| VirB9.1+Quil A |  | ns | **** | **** | ns | ns | **** | **** |
| VirB9.1/SV-100 | ns |  | **** | **** | ns | * | **** | **** |
| VirB9.2+Quil A | **** | **** |  | ns | **** | **** | * | * |
| VirB9.2/SV-100 | **** | **** | ns |  | **** | **** | ns | ns |
| VirB9.1/9.2+Quil A | ns | ns | **** | **** |  | ns | **** | **** |
| VirB9.1/9.2/SV-100 | ns | * | **** | **** | ns |  | **** | **** |
| SV-100 | **** | **** | * | ns | **** | **** |  | ns |
| Unimmunised | **** | **** | * | ns | **** | **** | ns |  |

**S1 Table.** Statistical analysis of ELISPOT results

| **Groups** | VirB9.1+Quil A | VirB9.1/SV-100 | VirB9.2+Quil A | VirB9.2/SV-100 | VirB9.1/9.2+Quil A | VirB9.1/9.2/SV-100 | SV-100 | Unimmunised |
| --- | --- | --- | --- | --- | --- | --- | --- | --- |
| VirB9.1+Quil A |  | ns | **** | **** | **** | **** | ns | ns |
| VirB9.1/SV-100 | ns |  | **** | **** | **** | **** | ns | ns |
| VirB9.2+Quil A | **** | **** |  | ns | ns | ns | **** | **** |
| VirB9.2/SV-100 | **** | **** | ns |  | ns | ns | **** | **** |
| VirB9.1/9.2+Quil A | **** | **** | ns | ns |  | ns | **** | **** |
| VirB9.1/9.2/SV-100 | **** | **** | ns | ns | ns |  | **** | **** |
| SV-100 | ns | ns | **** | **** | **** | **** |  | ns |
| Unimmunised | ns | ns | **** | **** | **** | **** | ns |  |

1. VirB9-1-specific ELISPOT assay results: *, p < 0.05; ****, p < 0.0001; ns = not significant.
2. VirB9-2-specific ELISPOT assay results: ****, p < 0.0001; ns = not significant.
